# Compartment-specific antibody correlates of protection to SARS-CoV-2 Omicron in macaques

**DOI:** 10.1016/j.isci.2024.110174

**Published:** 2024-06-04

**Authors:** Xin Tong, Qixin Wang, Wonyeong Jung, Taras M. Chicz, Ross Blanc, Lily J. Parker, Dan H. Barouch, Ryan P. McNamara

**Affiliations:** 1 Ragon Institute of Mass General, MIT, and Harvard, Cambridge, MA 02139, USA; 2 Beth Israel Deaconess Medical Center, Harvard Medical School, Boston, MA 02215, USA

**Keywords:** Health sciences, Biological sciences

## Abstract

Antibodies represent a primary mediator of protection against respiratory viruses. Serum neutralizing antibodies (NAbs) are often considered a primary correlate of protection. However, detailed antibody profiles including characterization of antibody functions in different anatomic compartments are poorly understood. Here we show that antibody correlates of protection against severe acute respiratory syndrome coronavirus 2 (SARS-CoV-2) challenge are different in systemic versus mucosal compartments in rhesus macaques. In serum, NAbs were the strongest correlate of protection and linked to spike-specific binding antibodies and other extra-NAb functions that create a larger protective network. In bronchiolar lavage (BAL), antibody-dependent cellular phagocytosis (ADCP) proved the strongest correlate of protection rather than NAbs. Within BAL, ADCP was linked to mucosal spike-specific immunoglobulin (Ig)G, IgA/secretory IgA, and Fcγ-receptor binding antibodies. Our results support a model in which antibodies with different functions mediate protection at different anatomic sites.

## Highlights

• Correlates of protection to SARS-CoV-2 Omicron are highly compartment specific
• Antibody effector functions are primary correlates of protection at infection site
• Mucosal boosting enhances IgA and functionally levered IgG in lower respiratory tract

## Graphical abstract

**Figure:**
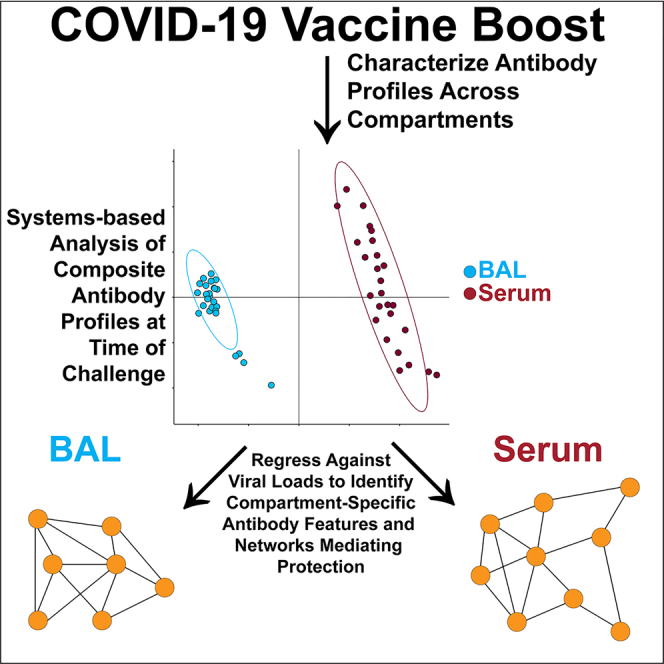

## Introduction

COVID-19 vaccines, which generate antibodies to the severe acute respiratory syndrome coronavirus 2 (SARS-CoV-2) spike protein, have shown remarkable success at attenuating severe disease. Neutralizing antibodies, which most commonly target the receptor binding domain (RBD) of spike, were identified as a correlate of protection against ancestral strains of SARS-CoV-2.^1^ However, as Omicron-lineage SARS-CoV-2 variants emerged, vaccine-/infection-acquired antibody neutralization was largely lost due to the high degree of antigenic shift within the RBD.^2^^,^^3^^,^^4^^,^^5^^,^^6^ Yet protection from disease in vaccinated individuals did not see a concomitant drop,^7^^,^^8^^,^^9^^,^^10^^,^^11^ signifying that immune mediators of protection other than neutralizing antibodies existed.

Beyond their capacity to neutralize, antibodies exert several non-neutralizing functions such as antibody-dependent opsinophagocytosis, antibody-dependent cellular cytotoxicity, and complement deposition.^12^ These functions are largely modulated by post-translational modifications to the crystallizable fragment (Fc) of antibodies which dictate their binding to Fc receptors (FcγR for immunoglobulin [Ig]G subclasses, FcαR for IgA subclasses, etc.) on the surface of immune cells. Previous reports have demonstrated that FcγR-binding antibodies can recognize highly diverged SARS-CoV-2 spikes and confer protection even when neutralization is lost.^13^^,^^14^^,^^15^ To that end, antibodies mediate protection against pathogens such as SARS-CoV-2 through a variety of functions.

It is unclear how antibody correlates of protection for COVID-19 are shaped in different anatomic compartments. In this study, we show the unexpected results that neutralizing antibodies are a strong correlate of protection in serum but are not a clear correlate of protection in mucosa. Instead, extra-neutralizing functions such as antibody-dependent cellular phagocytosis (ADCP) were the strongest correlate of protection in bronchiolar lavage (BAL) against SARS-CoV-2 Omicron challenge. This extra-neutralizing role was conserved across SARS-CoV-2 variant spikes, including the challenge strain. Our results support a model in which antibody correlates of protection against SARS-CoV-2 Omicron are different in different anatomic compartments.

## Results

### Identification of humoral features inversely correlated with SARS-CoV-2 viral loads by anatomic compartment

**Figure 1:**
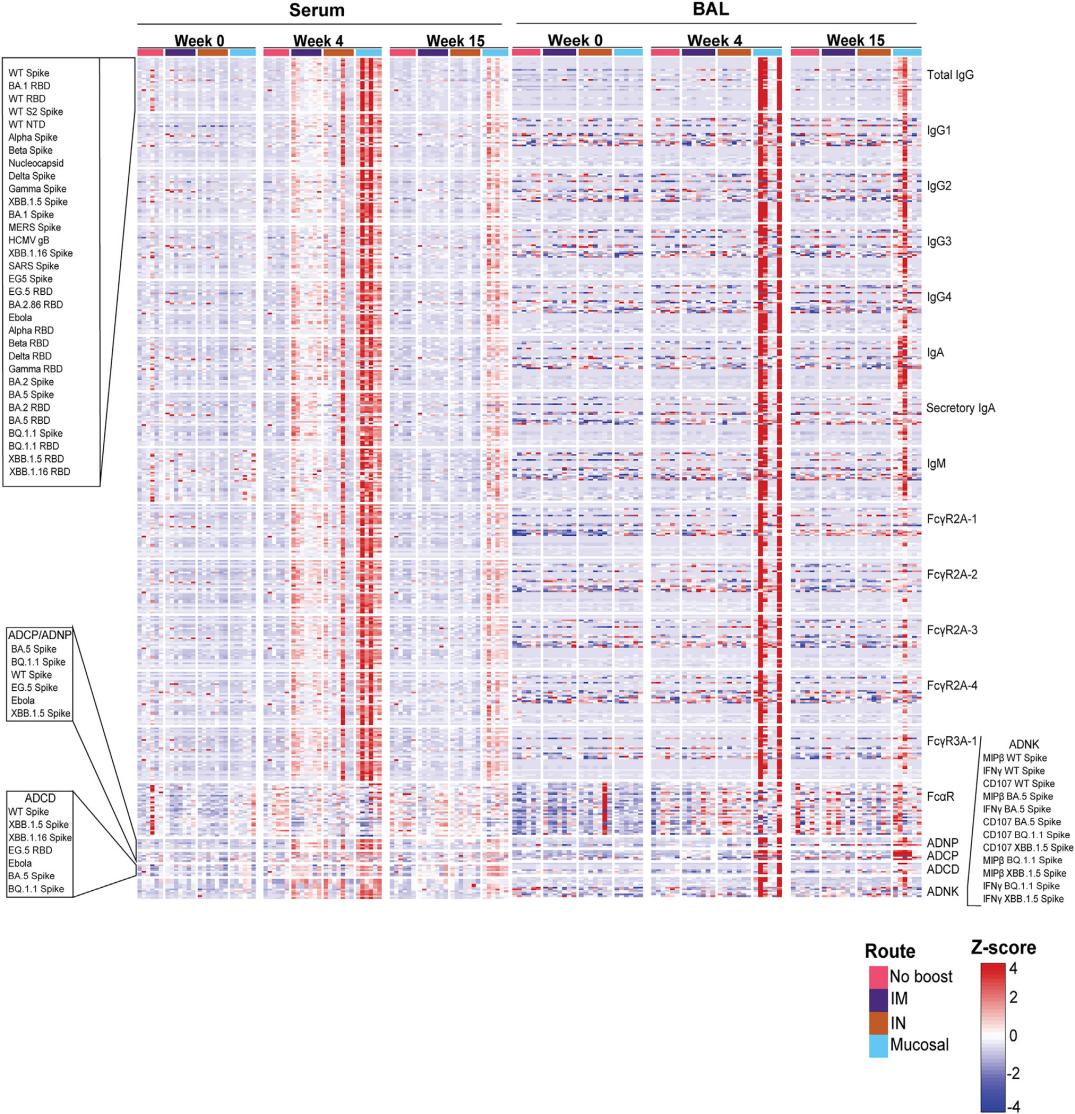
Full systems serology data array Serum (left) and BAL (right) antibody samples were assayed at week 0, week 4, and week 15 post-bivalent booster for binding to the antigens listed on the top left. Antibody subclass, isotype, Fc-binding, and functional outputs are shown on the right column. Route of administration and value legend is shown on the bottom right (IM = intramuscual and IN = intranasal). Callout boxes for ordering of antigens are shown on the left. All results were *Z* scored to account for differences in outputs. See also Figures S1 and S2.

**Figure 2:**
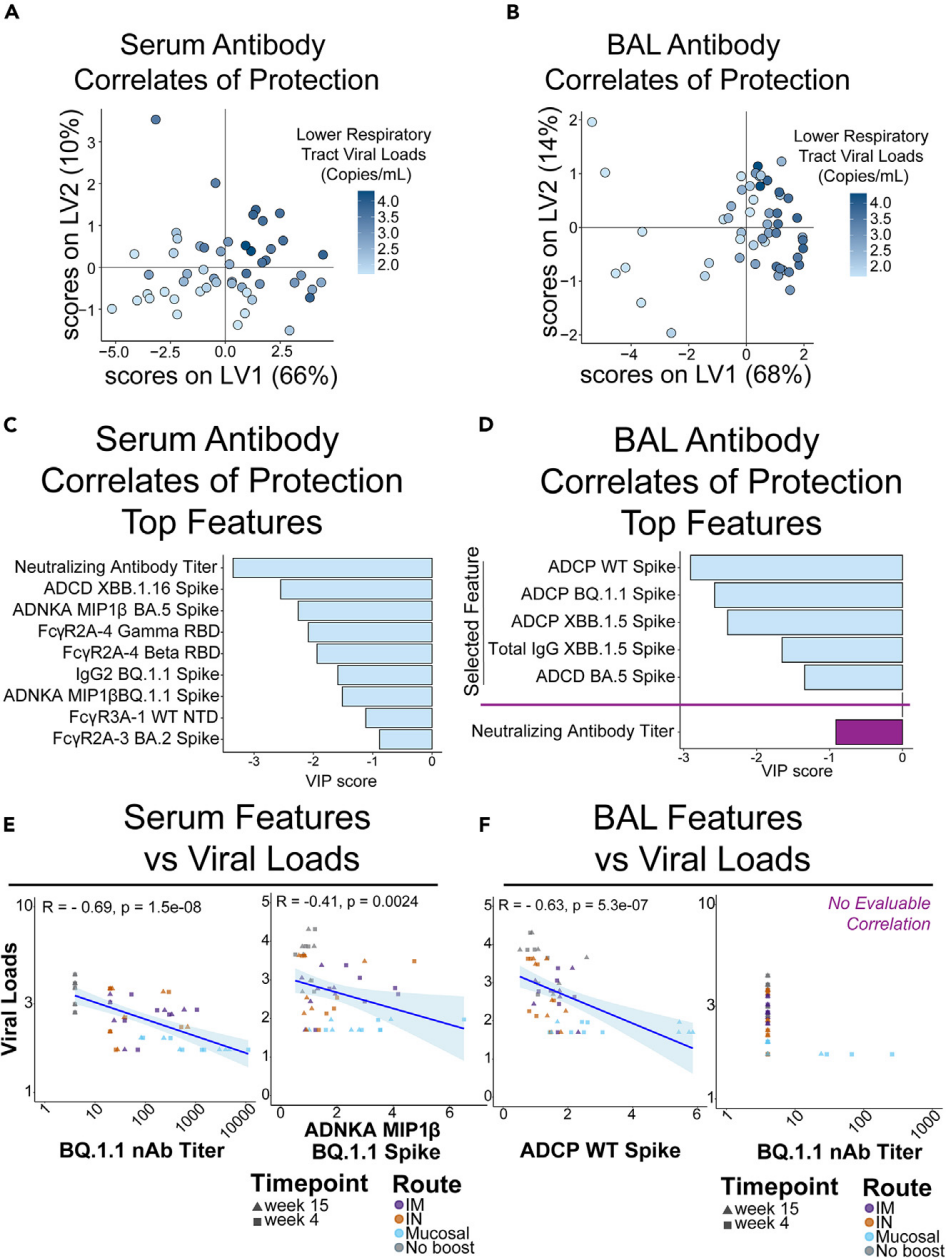
Defining antibody correlates of protection within serum and bronchiolar lavage (A) Partial least-squares regression (PLSR) model of serum antibody features of vaccinated and boosted non-human primates (NHPs) inversely correlated with viral loads within the lower respiratory tract. Heatmap gradient of viral loads is shown on the right. (B) PLSR model of BAL antibody features of vaccinated and boosted NHPs inversely correlated with viral loads within the lower respiratory tract. Heatmap gradient of viral loads is shown on the right. (C) Top serum antibody correlates of protection in the PLSR model. (D) Top BAL antibody correlates of protection in the PLSR model. Neutralizing antibody titer was not selected as a *bona fide* correlate in the BAL and was manually plotted in purple for comparison. (E) Validation of PLSR-selected serum correlates of protection. Viral loads were inversely correlated with (left) neutralizing antibody titer and (right) antibody-dependent natural killer cell activation (ADNKA) as measured by macrophage inflammatory protein 1 beta (MIP1β) production. Spearman’s R values and multiple comparisons adjusted *p*-values are shown. Trendline is shown with shaded areas being the 95% confidence interval. (F) Validation of PLSR-selected BAL correlates of protection. Viral loads were inversely correlated with (left) antibody-dependent cellular phagocytosis (ADCP) to BQ.1.1 spike (challenge strain), but not to neutralizing antibody titers. Spearman’s R values and multiple comparisons adjusted *p* values are shown only for the statistically significant ADCP. Trendline is shown with shaded areas being the 95% confidence interval. See also Figures S3 and S4.

Binding antibodies, Fcγ-receptor (FcγR) binding antibodies, neutralization titers, and Fc effector functions from serum- and lower respiratory tract-resident antibodies were quantified through systems serology (Figure 1, S1, and S2). A composite multivariate probable least-squares regression (PLSR) model was built to identify antibody features correlated with protection against viral loads across treatment groups. This was done for antibodies in serum (Figure 2A) and BAL (Figure 2B). Viral loads scattered on the latent variable 1 axis (LV1), which accounted for 66% and 68% of the total variance explained in the serum and BAL humoral profiles, respectively.

Distinguishing antibody features driving protection by compartment were identified. In the serum, neutralizing antibody titers, FcγR-binding antibodies, antibody-dependent natural killer cell activations (ADNKAs), and ADCP were all significant correlates of protection (Figure 2C, light blue bars). Interestingly, features driving protection in the BAL were focused on ADCP, IgG binding antibodies to Omicron XBB.1.5 spike, and antibody-dependent complement deposition (ADCD) (Figure 2D, light blue bars). Since neutralizing antibodies were not selected as a driver of protection in BAL, we separately added it for comparison relative to the other features (Figure 2D, purple bar).

To confirm these results, we plotted correlations of selected features with viral loads based on compartments. As expected, serum selected features at 4 and 15 weeks post-boost correlated with protection as defined by viral loads (Figures 2E and S3A). Likewise, BAL selected features correlated with protection at 4 and 15 weeks post-boost (Figures 2F left and S3B). A correlation could not be evaluated with BAL neutralizing antibodies as the majority of animals did not have neutralizing antibodies, and thus more data are required to evaluate BAL neutralizing correlates of protection (Figure 2F, right).

To exclusively look at the humoral profile immediately before SARS-CoV-2 BQ.1.1 challenge, the top PLSR-selected features were correlated against viral loads at week 15 post-boost. Similar to our previous observations, serum neutralizing antibody levels were significantly correlated with a reduction in viral loads within the lower respiratory tract at this time point (Figure S3C). Within the BAL, ADCP against both wild-type (WT) and the challenge strain BQ.1.1 remained significantly correlated with reduced viral loads (Figure S3D). Therefore, whether combining weeks 4 + 15 as a total “area under the curve” humoral features or exclusively looking humoral profiles immediately before virus challenge, neutralizing antibodies remained strong predictor of protection within serum while ADCP was the strongest predictor of protection at the mucosa. PLSR models for both serum and BAL-resident selected features as driving protection were validated against permutated labels and random features (Figure S4).

### BAL correlates of protection to SARS-CoV-2 are defined by binding, FcγR binding, and effector functions

To investigate how the humoral landscape operates at the systems level, we created constellation linkage profiles by compartment. To create these constellation linkage profiles by the compartment, humoral features driving protection in the specific compartment selected by PLSR were used as centering features, or nodes. Humoral features significantly correlated with these compartment-specific drivers of protection were then linked to the PLSR-selected feature (see STAR methods). Serum-resident antibody features driving protection were part of a large constellation of humoral features that included binding antibodies, FcγR-binding antibodies, and Fc-effector-mediated functions (Figure S5).

**Figure 3:**
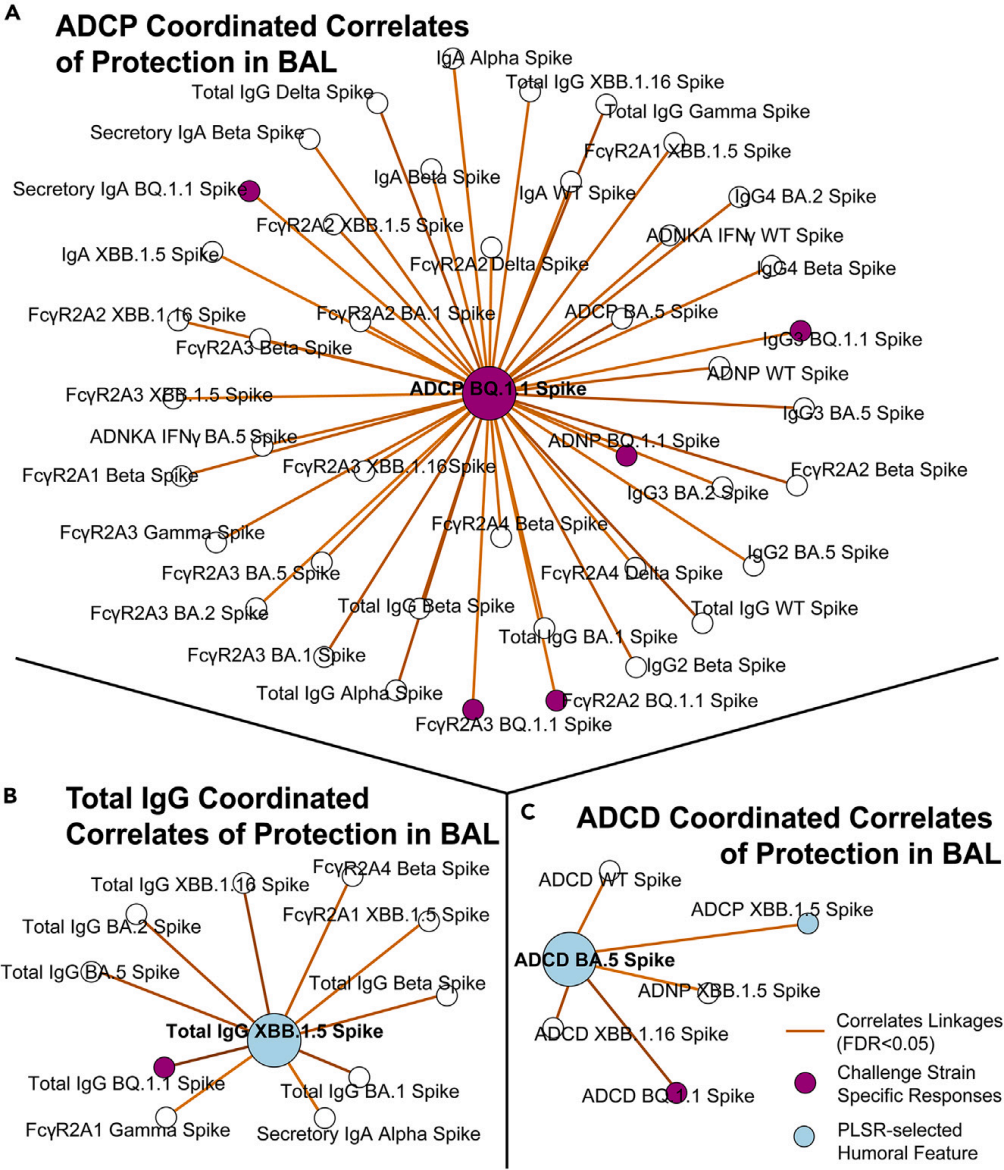
Antibody network of strongest correlates of protection in the BAL (A) ADCP to the challenge strain BQ. 1.1 spike (purple node), which was identified through PLSR as a correlate of protection against viral loads, was centered and co-correlating antibody features (R > 0.6, FDR *p* value <0.05) taken from the BAL are shown. (B) Same as (A), but for total IgG to XBB. 1.5 spike. (C) Same as (A), but for ADCD to BA.5 spike. Blue circles correspond to the selected features in Figure 1. Purple circles correspond to BQ.1.1 spike, which was the challenge strain. Legend is shown at the bottom right. Although features such as ADCD to XBB.1.16 were identified as protectors in both compartments, it was not highlighted in BAL correlates of protection as it was not a PLSR-selected feature. See also Figures S5 and S6.

ADCP to BQ.1.1 spike was identified as a protective feature within the BAL in this unbiased initial analysis. Humoral features significantly correlating with ADCP to BQ.1.1 were identified which included IgG subclasses, IgA, secIgA, and FcγR-binding antibodies, as well as to other effector functions (Figure 3A). A separate correlate of protection in the BAL was total IgG, and a constellation of co-correlating features was mapped showing other binding IgG and FcγR-binding antibodies across variants of concern (VOC) (Figure 3B). The last key correlate of protection identified was ADCD to XBB.1.5 spike. As expected, this node correlated with ADCD to other spike variants including Omicron sublineages (Figure 3C). The full constellation for BAL antibody correlates of protection is shown in Figure S6. We chose three constellations based on the function of the PLSR-selected feature (ADCP, ADCD, and Total IgG). We elected to use ADCP to BQ.1.1 as it was the challenge strain and thus represented the most relevant ADCP output. PLSR-selected features in the serum were not highlighted as they were decompartmentalized; however, we did note that several humoral features identified as protective features were present in both compartments.

For the BAL, neutralizing antibodies were neither selected as a driving feature of protection by the PLSR nor linked to features that were as shown in the constellation network. This is in stark contrast to the serum correlates of protection where neutralizing antibodies were selected by the PLSR and were thus a node in the serum constellation network. Within the serum, neutralizing antibodies were part of a complex network of co-correlating features, including to other PLSR-selected drivers of protection within the compartment.

### Mucosal boosting increases serum- and lower respiratory tract-resident humoral responses to divergent spikes

**Figure 4:**
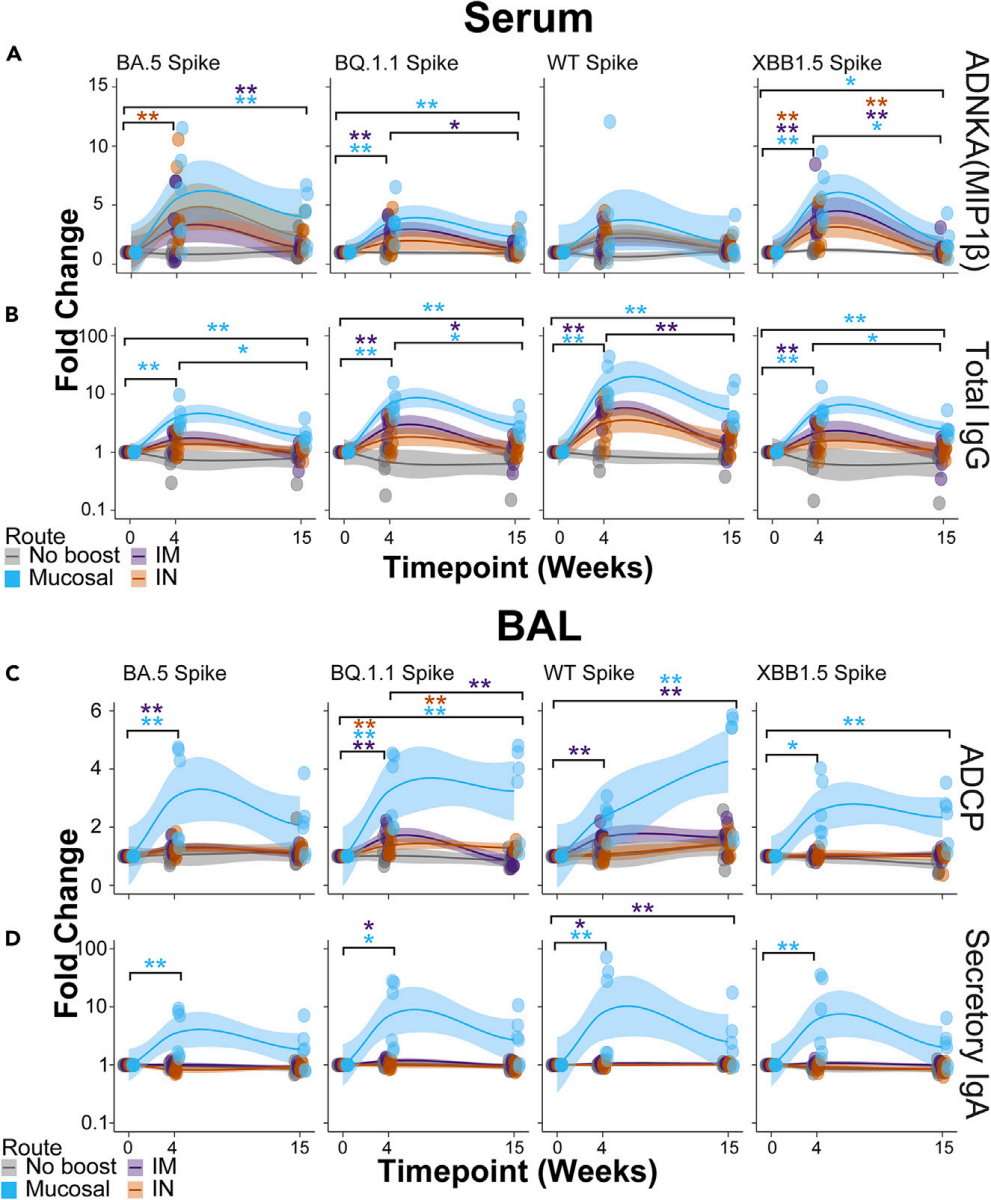
Mucosal boosting enhances serum and lower respiratory tract humoral responses to various SARS-CoV2 VOCs (A) Post-booster fold enhancements of serum-resident antibody-dependent natural killer cell activation (ANDKA) to the indicated spike variants as quantified by macrophage inflammatory protein 1 beta (MIP1β) production. ADNKA was selected as a key correlate of protection in Figure 1. Individual data points as well as moving averages are shown; the solid line is the mean and the shaded regions are the 95% confidence intervals. (B) Same as (A), but for the serum-resident networked feature of total IgG to the indicated spikes variants. Color scheme legend is shown at the bottom for the serum responses. (C) Post-booster fold enhancements of BAL-resident antibody-dependent cellular phagocytosis (ADCP) by monocytes to the indicated spike variants. ADCP was selected as a key correlate of protection in Figure 1. (D) Same as (C), but for the BAL-resident networked feature of secretory IgA to the indicated spike variants. Color scheme legend is shown at the bottom for the BAL responses. For all plots, fold enhancements were relative to the mean value of the group at the pre-boost time point, week 0. Shown here are the best-fit models of matched responses within 95% CI in regions shaded in corresponding colors. ∗ = *p* < 0.05, ∗∗ = *p* < 0.01; Wilcoxon test followed by FDR correction. See also Figures S7 and S8.

To define if correlates of protection were influenced by route of vaccine booster, we analyzed the identified correlates of protection using the delivery site as a variable. For ADNKA responses, non-boosted non-human primates (NHPs) showed no changes to any spikes at any time points. Intramuscular (IM)-boosted (purple) NHPs showed significantly enhanced serum ADNKA to BQ.1.1 and XBB.1.5 spikes. Intranasal (IN)-boosted (orange) NHPs likewise showed enhanced ADNKA responses but were significant for BA.5 and XBB.1.5 spike. Mucosal-boosted (blue) NHPs showed significant ADNKA responses to BA.5, BQ.1.1, and XBB.1.5 spike (Figure 4A). Significant expansions of ADNKA to WT spike were not observed for any groups, likely indicating that existing profiles to ancestral spike from previous vaccinations were still present. Binding IgG within the serum to the spikes strongly correlated with ADNKA. We thus assayed for total IgG binding to these spikes from the serum of the boosted NHPs. We found that total IgG to spike variants significantly increased for IM- and mucosal-boosted NHPs, but not for IN-boosted NHPs, after multiple comparisons adjustments (Figure 4B).

ADCP to several spikes was the strongest correlate of protection in the BAL. Similar to serum responses, we analyzed how ADCP responses were shaped by booster delivery site. Interestingly, mucosal-boosted (blue) NHPs showed the strongest and most consistent ADCP to spikes tested from BAL samples. IM-boosted (purple) NHPs also showed some significant ADCP expansions, but the magnitude of responses was much lower than mucosal-boosted NHPs. While some ADCP was observed from BAL samples from IN-boosted NHPs, only one spike showed significance after multiple test corrections (Figure 4C).

Within the mucosa, secIgA was identified as a significant co-correlate of protection along with ADCP of BQ.1.1 spike. Therefore, to validate our constellation analysis, we plotted responses of secIgA in the boosted NHPs. Similar to ADCP, secIgA was significantly boosted in NHPs that received a mucosal boost at 4 weeks post-boost. These secIgA levels waned but remained elevated for the mucosal-boosted NHPs at 15 weeks post-boost. IM-boosted NHPs showed low secIgA induction to some spikes, while IN-boosted NHPs did not show any significant secIgA increases for any spike assayed (Figure 4C). Therefore the correlation between secIgA and the identified BAL correlates of protection was strongly driven by booster site.

Other serum and BAL humoral profiles showed similar results, including functional assays (Figures S7 and S8). Within the BAL, mucosal-delivered boosts consistently yielded higher antibody levels and functional outputs. Collectively, these results confirm our machine learning approaches of classifying correlates of protection by compartment. These results are strongly influenced by the route of boosting.

### Mucosal boosting results in tight correlations between FcγR binding and effector functions

**Figure 5:**
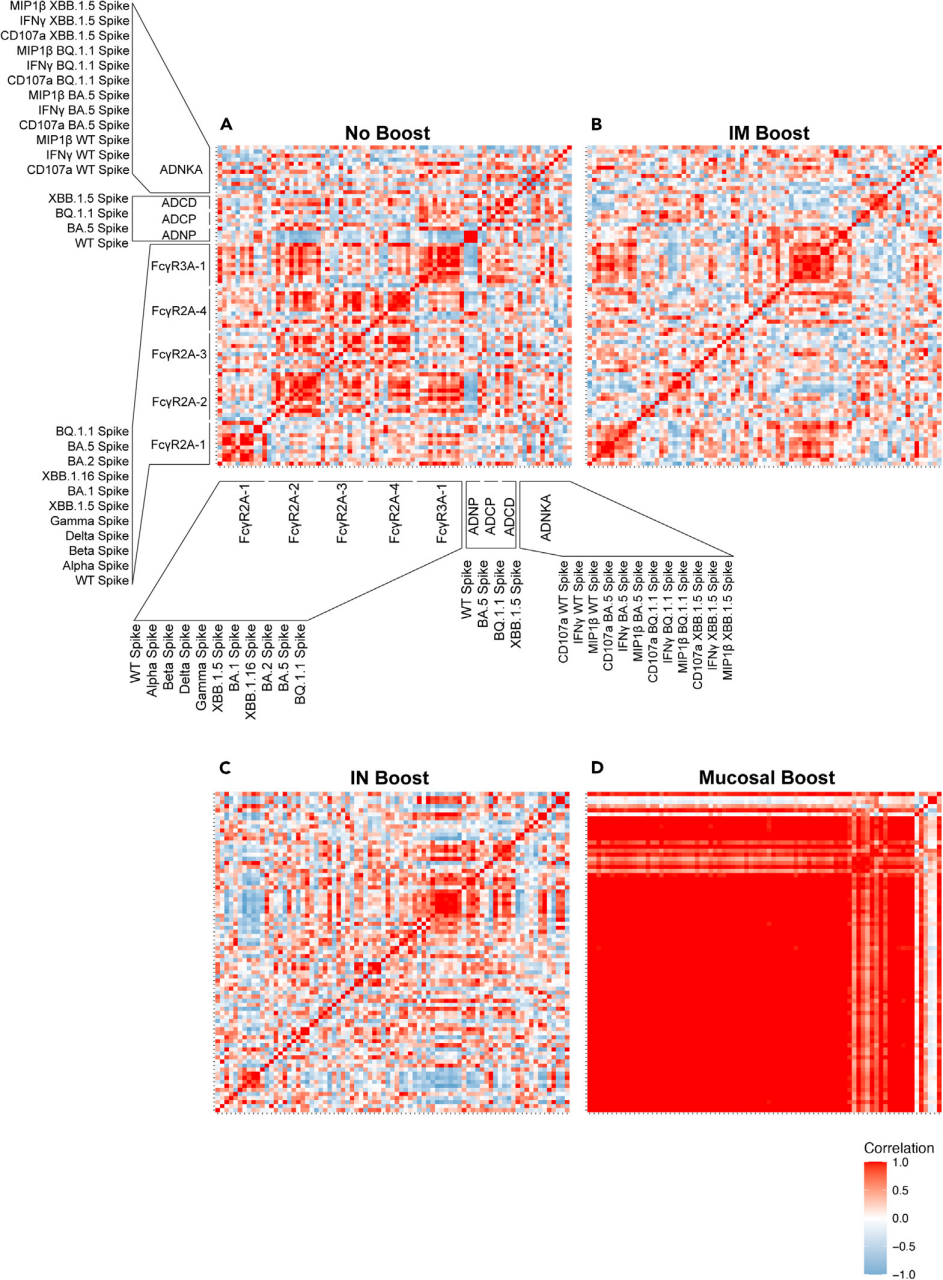
Mucosal boosting induces a highly correlated network of FcγR-binding antibodies and effector functions in the BAL (A) Correlation heatmaps were generated for FcγR-binding antibodies and effector-mediated functions at week 15 for the antigens used in this study for NHPs who received no boost. Callout boxes for the antigens used for the specific assays (FcγR-binding, ADCP, ADNP, ADCD, and ADNKA) and their orders are shown. (B) Same as (A), but for NHPs at week 15 that received an IM boost. (C) Same as (A), but for NHPs at week 15 that received an IN boost. (D) Same as (A), but for NHPs at week 15 that received a mucosal boost. Heatmap legend for the correlation coefficient is shown in the bottom right.

Given that effector-mediated functions such as ADCP, antibody-dependent neutrophil phagocytosis (ADNP), and ADNKA were disproportionally observed in mucosal-boosted NHPs, we asked if these functions were indeed correlated with FcγR-binding antibodies, particularly within the BAL. We conducted this analysis using week 15 time points only from BAL as this captured the humoral landscape and linkages at the time and site of challenge. Non-boosted NHPs showed non-specific and variable degrees of correlations between FcγR-binding antibodies and effector functions across VOC spikes, including the challenge strain BQ.1.1 (Figure 5A). Some scattered and loose correlations were identified for NHPs that were boosted through IN or IM within the BAL, but the overall architectures were uncoordinated (Figures 5B and 5C).

In stark contrast to the non-boosted, IM-, and IN-boosted NHPs, those that were mucosal boosted showed tight correlations across FcγRs and with functional outputs within the BAL immediately before challenge. This tight degree of correlation was almost completely ambivalent to the spike variant (Figure 5D). The sole exception was some natural killer (NK) cell readouts. However, overall, the FcγR-binding and functional output profile within the BAL were highly coordinated in mucosal-boosted NHPs. This provides further support that the humoral architecture within the BAL is highly leveraged for non-neutralizing functions that are linked with protection against viral loads.

Taken together, FcγR-binding antibodies and non-neutralizing functions are highly influenced by the route of booster administration. Moreover, humoral features driving protection within the BAL can act as a well-coordinated network with broad breadth of binding and functionality.

## Discussion

In this study, we report that antibodies with distinct functions in different anatomic compartments are correlates of protection against SARS-CoV-2 Omicron challenge in rhesus macaques. Our data support a model whereby mucosal antibody correlates of protection in the lower respiratory tract are primarily functional antibodies that mediate opsinophagocytosis, while serum antibody correlates of protection are primarily neutralization, binding, and non-neutralizing functions. Our data suggest that antibodies with Fc effector functions may be more important than currently appreciated at mucosal surfaces.

Previous work demonstrated that neutralizing antibodies were a correlate of protection against ancestral SARS-CoV-2 across several clinical trials.^1^ As SARS-CoV-2 adapted itself to the human population, VOCs emerged and neutralizing antibody capacity was progressively lost.^16^^,^^17^^,^^18^ This did not translate to a loss of clinical protection,^9^^,^^10^^,^^11^ indicating that correlates beyond neutralization existed. Previous reports have shown that FcγR-binding antibodies and their corresponding effector functions were required for protection against antigenically diverged spikes.^13^^,^^19^

We employed a systems serology approach to arrive at the conclusion that humoral profiles that correlate with protection are different in different anatomic compartments. Deep antibody profiling including a comprehensive analysis of antibody functions revealed that neutralizing antibody titers are the strongest correlate of protection in serum. This is in agreement with several reports showing that serum-resident neutralizing antibodies are strongly induced after vaccination and/or infection and are a correlate of protection.^20^^,^^21^^,^^22^^,^^23^^,^^24^^,^^25^^,^^26^^,^^27^^,^^28^ Our approach extends these findings to show that antibody correlates of protection are different at distinct anatomic sites. We show that antibody correlates of protection at the mucosa are enriched for effector functions such as opsinophagocytosis. These extra-neutralizing functions were most tightly linked to mucosa IgG and IgA/secIgA. Stimulation of IgA by different COVID-19 vaccine formulations has been shown.^29^ Our study shows that IgA can be stimulated by distinct delivery sites of the same vaccine formulation. IgA is known to be a potent neutralizer^30^^,^^31^^,^^32^ and a strong driver of opsinophagocytic function.^33^ Due to its polyfunctionality and mucosal localization, vaccine formulations and platforms have sought to enhance IgA responses.^29^^,^^34^^,^^35^^,^^36^^,^^37^ Our study supports the notion that vaccine delivery route can greatly impact mucosal IgA induction.

Much recent work has focused on characterizing mucosal protection against COVID-19 and other respiratory pathogens. This work extends on previous observations by McMahan et al.,^28^ Ying et al.,^38^ Hassan et al.,^39^ and other groups that have shown that targeted boosting at the mucosa can elicit superior protection against COVID-19.^34^^,^^35^^,^^36^^,^^37^^,^^40^ In our study, we show that this enhanced protection against COVID-19 is largely attributable to effector functions, particularly ADCP, at mucosal sites. This is in agreement with previous work by others that have shown that FcγR-binding antibodies were key determinants of protection against antigenically diverged spikes such as the Omicron sublineages and other sarbecoviruses.^13^^,^^14^^,^^15^^,^^19^ Also, recently Tong et al. showed that intranasal-delivered influenza vaccinations yielded a stronger effector function per antibody unit than intramuscular vaccination.^41^ It is well established that ADCC plays a key role in protection against influenza.^42^^,^^43^^,^^44^

Previous work has demonstrated that antibody recall responses to challenge are skewed to effector functions.^45^ These non-neutralizing responses were correlated with viral clearance shortly after infection.^46^ In the present study, we identify that protection in the BAL is linked with non-neutralizing functions post-boost. How long these effector functions persist after challenge and/or vaccination within the BAL is unclear. Future work at characterizing compartment-specific waning of effector functions similar to those published on waning of neutralization^47^^,^^48^^,^^49^^,^^50^ is needed.

In conclusion, our study demonstrates that antibody correlates of protection may be different in different anatomic compartments. In the lower respiratory tract, antibody Fc effector functions leveraged by IgG and IgA drive protection against SARS-CoV-2, whereas, in the serum, neutralizing antibodies drive protection. Further work characterizing how antibodies’ roles are influenced by their compartment can lead to vaccination strategies conferring multiple layers of protection. This is particularly important for emerging infectious diseases such as SARS-related coronaviruses.

### Limitations of the study

Our analysis to conclude that distinct compartments have unique humoral architecture relied on antibody binding, cellular response, and neutralization assays. Each of these has intrinsic limits of detection/sensitivity. For example, several animals had detectable neutralizing antibodies within the BAL and no viral loads; however, most did not have detectable neutralizing antibodies, while IgG and secIgA were readily detectable. We therefore cannot discount that the IgG and IgA present within the BAL may play a stronger role in neutralization. ADNKA is limited in its sensitivity and is a readout of low-affinity FcγR-signaling, namely through FcγIIIA (CD16a). How circulating antibody concentrations in the serum and BAL can activate ADNKA at different concentrations is unclear. Further work on harmonizing these distinct antibody profiling approaches is needed.

The use of human cells to characterize effector functions from NHPs has been done previously by others and us.^29^^,^^51^^,^^52^^,^^53^^,^^54^ How compartmentalization can affect the expression and/or induction of Fc receptors and their signaling is still being explored. It is known that copy numbers of FcγRIIIA and FcγRIIIB in humans can be linked to autoimmune diseases.^55^^,^^56^ Thus there exists a steady-state equilibrium to their expression. Future work into characterizing how stimuli affect the surface expression of these Fc receptors across compartments is warranted.

## STAR★Methods

### Key resources table

**Table:** 

REAGENT or RESOURCE	SOURCE	IDENTIFIER
**Antibodies**		

Anti-CD107a	BD Biosciences	RRID:AB_396136
Anti-CD3	BD Biosciences	RRID:AB_397038
Anti-CD16	BD Biosciences	RRID:AB_396864
Anti-CD56	BD Biosciences	RRID:AB_396853
Anti MIP-1β	BD Biosciences	RRID:AB_393549
Anti-IFNγ	BD Biosciences	RRID:AB_400425
Anti-guinea pig complement C3 goat IgG fraction, FITC	MP Biomedicals	RRID:AB_2334913
Goat anti-Mouse IgG Fc Cross-Adsorbed Secondary Antibody, PE	Thermo Fischer	RRID:AB_429715
Mouse Anti-rhesus IgG-PE (SB108a)	Southern Biotech	RRID:AB_2796071
Anti-rhesus IgG1 [7H11]	NHP Reagent Resource	RRID:AB_2819310
Anti-rhesus IgG2 [3C10]	NHP Reagent Resource	RRID:AB_2895607
Anti-rhesus IgG3 [2G11]	NHP Reagent Resource	RRID:AB_2819316
Anti-rhesus IgG4 [7A8]	NHP Reagent Resource	RRID:AB_2819322
Anti-rhesus IgA [9B9]	NHP Reagent Resource	RRID:AB_2819303
Anti-Secretory IgA	Invitrogen	RRID:AB_931432
Anti-rhesus IgM	Life Diagnostics	2C11-1-5

**Biological samples**		

LowTox Guinea Pig Complement	CedarLane Labs	Cat # CL4051

**Chemicals, peptides, and recombinant proteins**		

SARS-CoV-2 WT Spike	Sino Biological	40589-V08H4
SARS-CoV-2 BA1 RBD	Sino Biological	40592-V08H129
SARS-CoV-2 WT S2	Sino Biological	40590-V08B
SARS-CoV-2 WT NTD	Sino Biological	40591-V49H
SARS-CoV-2 Alpha Spike	Sino Biological	40589-V08B6
SARS-CoV-2 Beta Spike	Sino Biological	40589-V08B7
SARS-CoV-2 WT N	Sino Biological	40588-V08B
SARS-CoV-2 Delta Spike	Sino Biological	40589-V08B16
SARS-CoV-2 Gamma Spike	Sino Biological	40589-V08B10
SARS-CoV-2 XBB1.5 Spike	Sino Biological	40589-V08H45
SARS-CoV-2 BA1 Spike	Sino Biological	40589-V08H26
SARS-CoV-2 HCMV gB	Sino Biological	10202-V08H1
SARS-CoV-2 XBB1.16 Spike	Sino Biological	40589-V08H48
SARS-CoV-2 Alpha RBD	Sino Biological	40592-V08H82
SARS-CoV-2 SARS Spike	Sino Biological	40634-V08B
SARS-CoV-2 Beta RBD	Sino Biological	40592-V08H59
SARS-CoV-2 EG5 Spike	Sino Biological	40589-V08H55
SARS-CoV-2 BA2.86 Spike	Sino Biological	40589-V08H58
SARS-CoV-2 EG5 RBD	Sino Biological	40592-V08H151
SARS-CoV-2 Delta RBD	Sino Biological	40592-V08H91
SARS-CoV-2 Ebola Glycoprotein	Sino Biological	40459-V08H
SARS-CoV-2 Gamma RBD	Sino Biological	40592-V08H86
SARS-CoV-2 BA2 Spike	Sino Biological	40589-V08H28
SARS-CoV-2 BA2 RBD	Sino Biological	40592-V08H123
SARS-CoV-2 BA5 Spike	Sino Biological	40589-V08H32
SARS-CoV-2 BA5 RBD	Sino Biological	40592-V08H131
SARS-CoV-2 BQ1.1 Spike	Sino Biological	40589-V08H41
SARS-CoV-2 BQ1.1 RBD	Sino Biological	40592-V08H143
SARS-CoV-2 XBB1.5 RBD	Sino Biological	40592-V08H146
SARS-CoV-2 XBB1.16 RBD	Sino Biological	40592-V08H136
Rhesus soluble Fcγ2A-1	Duke University	Custom Order
Rhesus soluble Fcγ2A-2	Duke University	Custom Order
Rhesus soluble Fcy2A-3	Duke University	Custom Order
Rhesus soluble Fcy2A-4	Duke University	Custom Order
Rhesus soluble Fcγ3A-1	Duke University	Custom Order
LC-LC-Sulfo-NHS Biotin	ThermoFisher	Cat # A35358
Brefeldin A	Sigma Aldrich	Cat #B7651
GolgiStop	BD Biosciences	Cat # 554724
Streptavidin-R-Phycoerythrin	Prozyme	Cat # PJ31S

**Critical commercial assays**		

Fix & Perm Cell Permeabilization Kit (Medium B)	ThermoFisher	GAS002S100
Fix & Perm Cell Permeabilization Kit (Medium A)	ThermoFisher	GAS001S100
EasySep™ Direct Human Neutrophil Isolation Kit	Stemcell Technologies	19666
EasySep™ Human NK Cell Isolation Kit	Stemcell Technologies	17955
NHS-Sulfo-LC-LC Kit	ThermoFisher	21435
Zebra-Spin Desalting and Chromatography Columns	ThermoFisher	89882

**Experimental models: Cell lines**		

THP-1 monocytes	ATCC	RRID: CVCL_0006
Human Primary Natural Killer Cell Leukopack	StemCell	200–0092

**Software and algorithms**		

GraphPad Prism 8	GraphPad Software, Inc.	RRID:SCR_002798
iQue Forecyt 9.1	Sartorius	60028
R Studio V 6.0	R Project for Statistical Computing	RRID:SCR_000432
Flow Jo	BD Bioscience	RRID:SCR_008520
MATLAB ver. R2019a	MathWorks	RRID:SCR_001622

**Other**		

384-well HydroSpeed Plate Washer	Tecan	30190112
iQue Screener Plus	Intellicyt/Sartorius	11811
MagPlex Microspheres	Luminex MFG	MC12001-01 (Cataloged by region)
Green Fluorescent Neutravidin Microspheres	ThermoFisher	Custom Synthesis
Red Fluorescent Neutravidin Microspheres	ThermoFisher	Custom Synthesis
Scarlet Fluorescent Neutravidin Microspheres	ThermoFisher	Custom Synthesis
384-well HydroSpeed Plate Washer	Tecan	30190112
MagPlex Microspheres	Luminex MFG	MC12001-01 (Cataloged by region)
Green Fluorescent Neutravidin Microspheres	ThermoFisher	Custom Synthesis
Red Fluorescent Neutravidin Microspheres	ThermoFisher	Custom Synthesis
Scarlet Fluorescent Neutravidin Microspheres	ThermoFisher	Custom Synthesis
Systems Serology Dataset	GitHub	BDRPM_20240422
Existing dataset including neutralizing antibody titers and viral loads	McMahan et al.^28^	

### Resource availability

#### Lead contact

Further information and requests for resources and reagents should be directed to and will be fulfilled by the lead contact, Ryan P. McNamara (rpmcnamara@mgh.harvard.edu).

#### Materials availability

This study did not generate new unique reagents.

#### Data and code availability

• Raw systems serology data have been deposited at the Ragon Systems Serology homepage on GitHub under the accession: GitHub.com/RagonSystemSerology/DBRPM_20240422. This paper also analyzes existing, publicly available data. These accession numbers for the datasets are listed in the key resources table.
• This paper does not report original code. All scripts used for analysis can be found on the LoosC/systemsseRology on GitHub or can be obtained from the lead contact upon request
• Any additional information required to reanalyze the data reported in this paper is available from the lead contact upon request.

### Experimental model and study participant details

The experimental model for this study in rhesus macaques (*Macaca mulatta*) has been published previously and serum and BAL were collected for secondary use.^28^ Briefly, 4–8 year old rhesus macaques were administered two doses of Ad26.COV2.S vaccine IM and one IM boost with either Ad26.COV2.S or Ad26.COV2.351 (Beta Spike). The NHPs were given boosters with a bivalent Ad26.COV2.S + Ad26.COV2.S.529 (BA.1 Spike) by IM, IN, or mucosal (intratracheal) route (6–8 animals/group). Serum and BAL collections were done at week 0 (pre-boost), week 4, and week 15 post-boost. Animals were subsequently challenged at week 16 with SARS-CoV-2 Omicron BQ.1.1 with 2E+6 PFU through intratracheal delivery. Viral loads were monitored and quantified in the lower respiratory tract (Figure S1A). As described in the previous study,^28^ all animal study protocols were designed and conducted in compliance with all relevant local, state, and federal regulations and were approved by the Bioqual Institutional Animal Care and Use Committee (IACUC).

### Method DETAILS

#### Ig Subclassing/Isotyping and FcγR binding

Levels of antigen-specific antibody subclasses/isotypes and Fc-gamma receptor (FcγR) interaction were evaluated via the multiplexing Luminex microsphere-based assay, as previously described.^57^ Antigens of target were covalently linked to carboxyl group-labeled MagPlex microspheres (Luminex) through NHS-ester linkages using Sulfo-NHS and EDC (Thermo Fisher). Serum and BAL samples were diluted (serum isotypes/subclasses and FcγR binding: 1:250, BAL isotypes/subclasses and FcγR binding: 1:25) and added to the antigen-coupled microspheres to form the immune complexes in 384-well plates, and subsequently incubated at 4°C overnight, shaking at 750 rpm. After incubation, plates were washed with the washing buffer containing 0.1% BSA and 0.02% Tween 20 in PBS. Following the wash step, antibody isotype/subclass-specific mouse anti-rhesus antibodies (NHP Reagent Resource) were added to the immune complexes and incubated at room temperature for 1 h. Following a second wash step, the anti-mouse IgG Fc cross-adsorbed secondary antibody (PE, Thermo Fisher) was added to detect the anti-rhesus antibodies with fluorescence. For measurement of FcγR binding activities, Avi-tagged *Rhesus macaque* FcγRs (Duke Human Vaccine Institute) were biotinylated using BirA500 kit (Avidity) per manufacturer’s instructions and tagged with streptavidin-PE. The PE-labeled FcγR was subsequently incubated with the immune complexes for 2 h at room temperature. The plates were then washed and subject to Flow Cytometry measurements (iQue, Intellicyt) to determine the median fluorescence intensity (MFI). All Luminex experiments were conducted in duplicate, and the final results reported show the average values of the duplicates. The reagents and materials used are listed in the key resources table.

#### Antibody-dependent cellular phagocytosis and neutrophil phagocytosis

ADCP and ADNP experiments were performed as previously described.^58^^,^^59^ Briefly, antigen proteins of the target were biotinylated using the EZ-linkSulfo-NHS-LC-LC-Biotin kit (Thermo Fisher), then coupled to the fluorescent neutravidin beads (Thermo Fisher, F8776). The bead-antigen conjugates were incubated with diluted serum and BAL samples (serum: 1:100, BAL: 1:10) for 2 h at 37°C. The unbound antibody was removed by washing buffer. The immune complexes were then incubated overnight with cultured THP-1 cells (ADCP), or for 1 h with primary neutrophils isolated from human whole blood (ADNP) using negative selection (Stemcell). Treated THP-1 cells were subsequently washed and fixed in 4% paraformaldehyde (PFA), while the treated neutrophils were washed, stained for CD66b+ marker (Biolegend), and fixed in 4% (PFA) prior to flow cytometry analysis. A phagocytosis score for THP-1 or neutrophil was eventually determined as (% cells positive × Median Fluorescent Intensity of positive cells). Flow cytometry was performed with an iQue (IntelliCyt) instrument and population measurements were conducted using IntelliCyt ForeCyt (v8.1). The reagents and materials used are listed in the key resources table.

#### Antibody-dependent complement deposition (ADCD)

ADCD assays were designed and performed as previously described.^60^ Antigens of target were covalently linked to the carboxyl group-labeled MagPlex microspheres (Luminex) through NHS-ester linkages using Sulfo-NHS and EDC (Thermo Fisher) as described for Luminex. Diluted serum and BAL samples (serum: 1:50, BAL: 1:10) were incubated with coupled antigens for 2 h at 37°C to form immune complexes in 384-well plates. Plates were washed and incubated with lyophilized guinea pig complement (Cedarlane) diluted in gelatin veronal buffer with calcium and magnesium (Sigma Aldrich) for 20 min at 37°C. The deposition of C3 complement component was evaluated by an anti-guinea pig C3 FITC detection antibody (MpBio). Fluorescent intensity was acquired using an iQue Flow Cytometer (Intellicyt). The antibody-specific complement C3 deposition is calculated as the median fluorescence intensity of FITC. All ADCD experiments were conducted in duplicate, and final values were reported as average of the duplicates. The reagents and materials used are listed in the key resources table.

#### Antibody-dependent natural killer cell (NK) activation (ADNKA)

ADNKA assays were designed and performed as described previously.^61^ ELISA plates were coated 3 μg/mL of selected antigen and incubated at 4°C overnight. The coated plates were washed with PBS and blocked with 5% bovine serum albumin (BSA) for 2 h at 37°C. Natural Killer (NK) cells were isolated from Leukopaks (Stemcell Technologies) using EasySep Human NK Cell Isolation Kit (Stemcell Technologies). The isolated NK cells were incubated overnight at 37°C 5% CO2 in R10 (RPMI-1640 (Sigma Aldrich) media supplemented with 10% fetal bovine serum (FBS) (Sigma Aldrich), 5% penicillin/streptomycin (Corning, 50 μg/mL), 5% L-glutamine (Corning, 4 mM), 5% HEPES buffer (pH 7.2) (Corning, 50 mM) supplemented with 2 ng/mL IL-15. The ELISA plates were washed, and diluted serum and BAL samples (serum: 1:40, BAL: 1:10) were added to plates for 2 h at 37°C to form immune complexes. After wash, NK cells were added to plates at a concentration of 2.5E+5 cells/mL in R10 media supplemented with anti-CD107a–phycoerythrin (PE)–Cy5 (BD Biosciences, lot # 0149826, 1:1000 dilution), brefeldin A (10 μg/mL) (Sigma-Aldrich), and GolgiStop (BD Biosciences). The NK cells were incubated with immune complexes for 5 h at 37°C. The incubated NK cells were stained for cell surface markers with anti-CD3 Pacific Blue (BD Biosciences, clone G10F5)), anti-CD16 allophycocyanin (APC)-Cy5 (BD Biosciences, clone 3G8), and anti-CD56 PE-Cy7 (BD Biosciences, clone B159) for 15 min at room temperature. The washed NK cells were then fixed with PermA (Life Technologies), permeabilized with PermB (Life Technologies), and labeled with anti-MIP-1β PE (BD Biosciences) and anti-IFNγ FITC for 15 min at room temperature. Fluorescent intensity was measured using iQue Cytometer (Intellicyt). NK cells were gated as CD56+/CD16+/CD3-and the NK activation was evaluated as the percentage of NK cells positive for CD107a, IFNγ, or MIP-1b. All assays were performed with at least two healthy donors and the results shown here report the average of the donors. The reagents and materials used are listed in the key resources table. A gating strategy figure can be found in Figures S1B–S1H.

#### Pseudovirus neutralization assay

The Nab data represent lentivirus-based pseudovirus neutralization assays that have been previously published and incorporated in this study for comparison.^28^

### Quantification and statistical analysis

#### Compartment-specific partial least-squares regression analysis (PLSR)

Partial Least Square Regression (PLSR) model was utilized to determine the best feature combination that describes peak viral load within the lower respiratory tract. Systems serology data including Ig isotype-, Ig subclass-, and FcγR-binding, as well as functional outputs ADCD, ADNP, and ADNKA, were regressed against peak viral loads within the lower respiratory tract of all animals. Neutralizing antibody titers from McMahon et al. were also incorporated into this analysis. Regressions were initially plotted as ambivalent to the route of booster delivery. The features that contributed most to the regression model were selected by LASSO (Least Absolute Shrinkage and Selection Operator). Features that were selected from more than 90% (Serum samples) or 80% (BAL samples) of 100 LASSO selections were finally selected for PLSR. PLSR model performance was determined by 5-fold cross-validations that were repeated 40 times. In addition, control models with random features or permuted output labels were built 25 times, whose accuracies were compared with the accuracy of the original model with 5-fold cross-validations. The LASSO-selected features were shown in the order of Variable Importance in Projection (VIP). For the BAL, neutralizing antibody levels regression against viral loads were manually plotted within the VIP. The compartment PLSR analysis was done for both time of challenge (week 15 post-booster only), as well as an “area under the curve” (AUC, 4 + 15 weeks post-booster).

#### Univariate correlations of PLSR

To further validate the PLSR, univariate correlations against viral loads for the selected humoral features were plotted. Viral loads are the log 10 of RNA copies/mL detected within the lower respiratory tract, and humoral features are plotted as the fold induction over baseline (week 0). This was done for both time of challenge (week 15 post-booster only) as well as an AUC (4 + 15 weeks post-booster). For the AUC univariate correlations, route of booster delivery is shown by the color of the symbol and time since booster is shown by the shape of the symbol. For the time of challenge univariate correlations, the route of booster delivery is shown by the color of the symbol. Spearman’s correlation coefficient (R) for each comparison is shown with an FDR adjusted *p*-value.

#### Compartment-specific constellation networks of protection

Humoral correlates with the PLSR-identified and validated correlates of protection were defined as having a Spearman’s correlation >0.6 with a false discovery rate (FDR) < 0.05 to the features. PLSR-selected humoral correlates of protection are shown in blue nodes, and features against the challenge strain BQ1.1 are shaded in purple. For these co-correlates networking (constellation networks), humoral features that were selected as significant drivers of protection by the PLSR were used as the centering features. Other humoral features that were correlated with that specific PLSR-selected feature (*p* < 0.05 after FDR correction, and an R value >0.6) were plotted as a linked constellation network. The length of the link does not contain any statistical information beyond achieving correlation with the above threshold.

#### Univariate comparisons

For univariate analysis, the “rstatix” R package was used, and two-sided Wilcoxon tests were performed to determine if data from different timepoints (week 0 vs. week 4, week 4 vs. week 15, and week 0 vs. week 15) significantly differed. *p* values were then corrected for multiple comparisons through FDR correction. Resulting *p*-values <0.05 were considered as significant. For all groups, baseline antibody levels were standardized to 1 to quantify fold changes. Moving averages from week 0, week 4, and week 15 post-booster were modeled and plotted showing the mean and 95% confidence intervals. Individual data points are also shown.
